# Targeting Reciprocally Connected Brain Regions Through CAV-2 Mediated Interventions

**DOI:** 10.3389/fnmol.2019.00303

**Published:** 2019-12-10

**Authors:** Sarah Morceau, Robin Piquet, Mathieu Wolff, Shauna L. Parkes

**Affiliations:** ^1^CNRS, INCIA, UMR 5287, Bordeaux, France; ^2^Université de Bordeaux, INCIA, UMR 5287, Bordeaux, France

**Keywords:** neural circuits, DREADD, prefrontal cortex, thalamus, insular cortex, basolateral amygdala

## Abstract

An important issue in contemporary neuroscience is to identify functional principles at play within neural circuits. The reciprocity of the connections between two distinct brain areas appears as an intriguing feature of some of these circuits. This organization has been viewed as “re-entry,” a process whereby two or more brain regions concurrently stimulate and are stimulated by each other, thus supporting the synchronization of neural firing required for rapid neural integration. However, until relatively recently, it was not possible to provide a comprehensive functional assessment of such reciprocal pathways. In this Brief Research Report, we highlight the use of a chemogenetic strategy to target projection-defined neurons in reciprocally connected areas through CAV-2 mediated interventions in the rat. Specifically, we targeted the bidirectional pathways between the dorsomedial prefrontal cortex (dmPFC) and the mediodorsal thalamus, as well as those connecting the insular cortex (IC) and the basolateral complex of the amygdala (BLA). These data showcase the usefulness of CAV-2-related strategies to address circuit-level issues. Moreover, we illustrate the inherent limitation of Cre-dependent adeno-associated virues (AAVs) with “leaked” expression of the gene of interest in the absence of Cre and highlight the need for appropriate control conditions.

## Introduction

A fundamental challenge for systems neuroscience is to connect structure to function. This becomes more difficult when considering distributed neural circuits with complex connectivity. One particularly intriguing feature of many distributed neural circuits is the reciprocity of the connections between two of their key elements. Functionally, an influential account posits that this organization enables re-entry, a process whereby two or more brain regions concurrently stimulate and are stimulated by each other, thus supporting the synchronization of neural firing required for rapid neural integration (Edelman and Gally, 2013). This account assumes global functions for reciprocal pathways such as categorizing sensory inputs, manipulating mental constructs and generating motor commands (Edelman and Gally, 2013; Wolff and Vann, 2019) but does not address the directionality of the exchanges within such “re-entrant” pathways. The aim of this Brief Research Report is to highlight the versatility of a CAV2-mediated strategy to target projection-defined neurons in reciprocally connected areas. We provide two such examples by using the CAV2-Cre vector and a Cre-dependent adeno-associated virus (AAV) carrying an inhibitory Designer Receptor Activated by Designer Drugs [DREADDs; hM4D(Gi); Armbruster et al., 2007] to target a thalamocortical circuit and the reciprocal connections between the gustatory portion of the insular cortex (IC) and the basolateral complex of the amygdala (BLA; Sripanidkulchai et al., 1984; McDonald, 1998; Yamamoto, 2006). Finally, we illustrate the known problem of Cre-independent transgene expression (e.g., Sjulson et al., 2016) at commonly used titrations to highlight the need for systematic control conditions in these types of interventions.

## Methods

**Figure 1:**
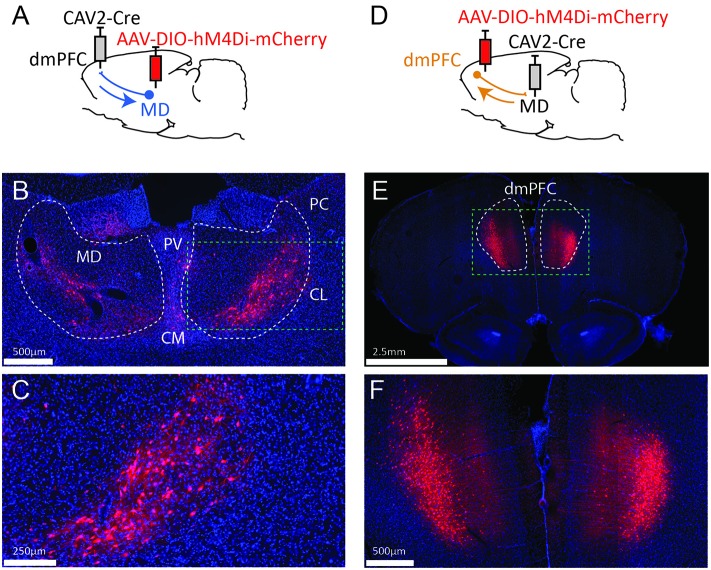
Strategy to target projection-defined thalamic **(A)** or cortical **(D)** neurons. The resulting labeling appears consistent with the currently known connectivity both in the MD **(B,C)** and the dmPFC **(E,F)**. The green dotted lines in **(B,E)** correspond to the areas magnified in **(C,F)**, respectively. PV, paraventricular nucleus; CM, centromedial nucleus; PC, paracentral nucleus; CL, centrolateral nucleus; MD, mediodorsal thalamus; dmPFC, dorsomedial prefrontal cortex (A32d). Images were captured using a Nanozoomer slide scanner (Hamamatsu Photonics) and the NDP.view 2.0^®^ freeware (Hamamatsu Photonics).

**Figure 2:**
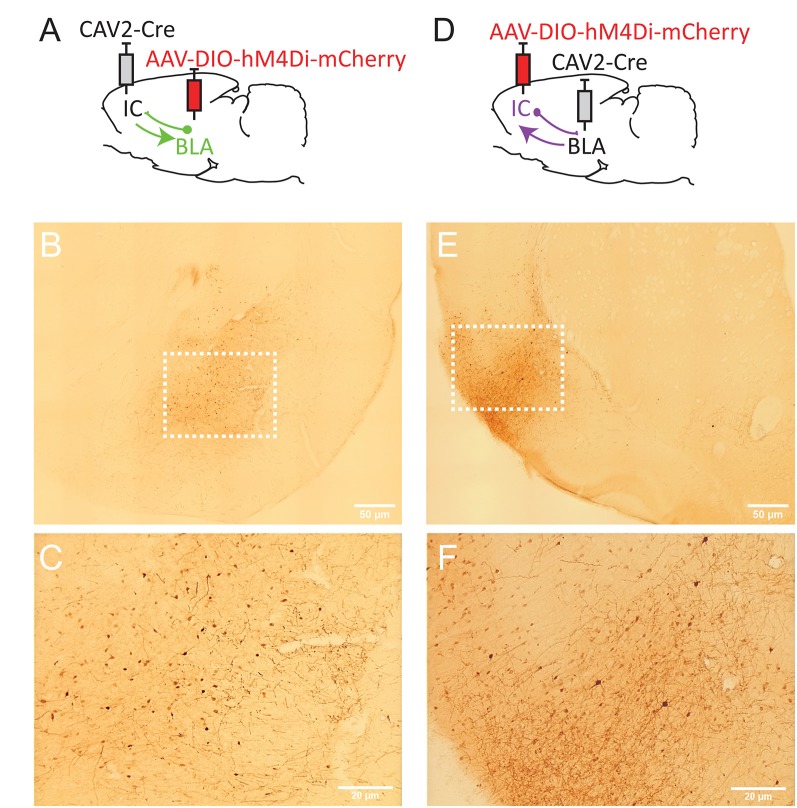
Strategy to target projection-defined amygdala **(A)** or insular cortex (IC; **D**) neurons. mCherry labeling in the basolateral complex of the amygdala (BLA; **B,C**, Bregma −2.76 mm) and the IC (**E,F**; Bregma +1.92 mm). The white dotted lines in **(B,E)** correspond to the areas magnified in **(C,F)**, respectively. Images were captured using a Leitz laborlux S microscope (10× objective) equipped with a Nikon 3CCD color camera.

We used an AAV carrying a floxed inhibitory DREADD receptor (hM4Di; Armbruster et al., 2007) and the retrograde CAV-2 vector (Junyent and Kremer, 2015) carrying the Cre recombinase to selectively express the inhibitory receptor in neurons based on their anatomical connectivity. First, we targeted the reciprocal connections between the mediodorsal thalamus (MD) and the dorsomedial prefrontal cortex (dmPFC), as shown in Figures 1A,D. Then, in a separate group of rats, we targeted the bidirectional pathways between the IC and the BLA, as shown in Figures 2A,D.

### Animals and Housing Conditions

Twenty-four male Long Evans rats weighing 275 g to 300 g at surgery were obtained from Centre d’Elevage Janvier (France). Housing conditions were the same as previously described (Alcaraz et al., 2018; Parkes et al., 2018), in accordance with current laws and policies (French Council directive 2013-118, February 1, 2013 and European directive 2010-63, September 22, 2010). The experimental protocols received approval #5012053-A from the local Ethics Committee on December 7, 2012. Six rats were used to assess connections between the MD and the dmPFC (three for MD-to-dmPFC and three for dmPFC-to-MD connections) and six others to assess the insular-BLA circuit (three for IC-to-BLA and three for BLA-to-IC connections). Another set of 12 rats was used to generate the different control conditions (three for each titer conditions for single AAV injections and another three to test the IC-to-BLA projection with the most diluted AAV condition).

## Surgery

Rats were anesthetized and prepared for stereotaxic surgery, as previously described (see Alcaraz et al., 2018; Parkes et al., 2018). For the thalamocortical circuit, CAV-2 and AAV were pressure injected (Picospritzer, General Valve Corporation, Fairfield, NJ, USA) into the brain through a glass micropipette (outside diameter: around 100 μm) and polyethylene tubing. In all cases, the needle was left in place 5 min after injection before slow retraction.

To target the MD-to-dmPFC pathway, 1 μl of 1 × 10^9^ genomic copies/μl (gc/μl) of CAV2-Cre (Biocampus PVM, Montpellier, France) was injected bilaterally in the dmPFC at the following coordinates (in mm from Bregma): AP +3.2, ML ±0.6, DV −3.4 mm. In the same surgery session, 1 μl of 1 × 10^9^ gc/μl of AAV8-hSyn-DIO-hM4Di-mCherry (UNC Vector Core, Chapel Hill, NC, USA) was injected bilaterally in MD at the following coordinates: AP −2.6, ML ±0.7 and DV −5.6. To target the dmPFC-to-MD pathway in a separate group of rats, virus injections were reversed, i.e., CAV-2 in the MD and AAV in the dmPFC. All injection parameters were the same, except for the mediolateral coordinates of AAV injection in the dmPFC, which were set at ±0.8 mm, to preferentially target cortical layers V and VI that project to the MD.

For the temporocortical circuit, CAV2 and AAV were microinjected (UMP3-1 and Micro4 Controller, World Precision Instruments) *via* a 10 μl NanoFil syringe with a blunt, 33 G needle. To target the BLA-to-IC pathway (Figure 2A), 0.25 μl of 1.21 × 10^12^ gc/μl of AAV8-hSyn-DIO-hM4Di-mCherry (Addgene plasmid, Viral Vector Production Unit, Universitat Autonoma de Barcelona, Spain) was injected bilaterally in BLA at two sites (in mm from Bregma): AP −2.0, ML ±4.6, DV −8.7 and AP −3.0, ML ±5.0, DV −8.7. In the same surgery session, 1 μl of 1 × 10^9^ gc/μl of CAV2-Cre (Biocampus PVM, Montpellier, France) was injected bilaterally at two sites in IC (in mm from Bregma): AP +0.7, ML ±5.5, DV −7.4 and AP +1.7, ML ±5.0, DV −7.0. To target the IC-to-BLA pathway, virus injections were reversed, i.e., CAV-2 in BLA and AAV in IC (Figure 2B).

To determine the extent of Cre-independent viral expression, we also injected AAV8-hSyn-DIO-hM4Di-mCherry (Addgene, Cambridge, MA, USA) alone at three titrations in another set of rats. The IC-to-BLA pathway was selected for this control condition. We therefore injected 1 μl of 1.21 × 10^12^ gc/μl, 4.8 × 10^11^ gc/μl or 4.8 × 10^10^ gc/μl at two sites in the IC. We then tested whether the weakest AAV titration (4.8 × 10^10^ gc/μl) was still effective in promoting Cre-dependent expression by injecting the AAV8 in the IC and the CAV2-Cre in the BLA (0.25 μl of 1.21 × 10^10^ gc/μl injected at two sites).

### Histology

For optimal viral expression, rats were perfused transcardially >1 month after surgery with 4% paraformaldehyde in 0.1 M phosphate buffer (PFA). Brains were kept in the same PFA solution overnight then, 40 μm sections were cut using a vibratome. Immunochemistry [fluorescent and non-fluorescent (DAB)] was performed to enhance mCherry staining. The fluorescent staining protocol is described in detail in Alcaraz et al., 2018. Briefly, sections were rinsed in 0.1 M phosphate-buffered saline (PBS), incubated in a blocking solution for 1 h, and then incubated with rabbit anti-RFP primary antibody (1:200 in blocking solution, Clinisciences, PM005) at 4°C for 48 h. The sections were then rinsed in PBS and placed in a bath containing goat anti-rabbit coupled to DyLight^®^ 549 (1:200 in PBS, 2 h; Jackson ImmunoResearch, 111-025-003). Following rinses in PBS, sections were incubated in Hoechst solution for neuronal counterstaining (1:5,000 in PBS, 15 min, bisBenzimide H 33258, Sigma, B2883). Finally, sections were rinsed, mounted onto gelatin-coated slides and coverslipped with the anti-fading reagent Fluoromount^®^ G (SouthernBiotech, 0100-01).

For non-fluorescent staining, floating sections were prepared by rinsing in 0.1 M PBS with 0.3% Triton X-100 (PBST; 5 × 5 min), and then in 0.5% H_2_O_2_ in 0.1 M PBST for 30 min. Following further rinsing in PBST, sections were incubated with rabbit anti-RFP (1:1,000 in PBST, Clinisciences, PM005) for 24 h at room temperature (RT). Then, the secondary antibody was applied (goat anti-rabbit biotinylated antibody, 1:1,000 in PBST containing 1% goat serum) and sections were incubated for 2 h at RT. Slices were then rinsed in PBST, incubated in avidine-biotinylated complex kit for 2 h at RT, rinsed in PBST and 0.05 M Tris buffer. Staining was revealed using 3,3′-Diaminobenzidine (DAB) solution (10 mg DAB + 50 ml 0.05 M Tris + 20 μl 30% H_2_O_2_) for 10 min. Finally, sections were rinsed, mounted and coverslipped with Eukitt (Sigma–Aldrich, St. Louis, MO, USA).

## Results

### Connections Between the Medial Prefrontal Cortex and the Mediodorsal Thalamus

To target dmPFC-projecting MD cells, we injected an AAV carrying a floxed hM4Di receptor expression cassette in the MD and the retrograde CAV2-Cre in the dmPFC (Figure 1A). As a result, only thalamic cells projecting to the dmPFC should be infected by both vectors and therefore express mCherry and hM4Di. As shown in Figures 1B,C, the actual observations were consistent with this expectation as mCherry expression was more evident in the lateral portion of the MD, in agreement with our current knowledge of these thalamocortical projections (Groenewegen, 1988; Alcaraz et al., 2016). mCherry expression was also visible to some degree in adjacent dmPFC-projecting thalamic areas such as the intralaminar group (paracentral and centro-lateral nuclei, mostly) and, to some extent, the centro-medial and the paraventricular nuclei. In some cases, fluorescence was also observed in the habenula. Next, injection sites for the viral construct were reversed in a distinct set of rats (Figure 1D). This manipulation produced heavy labeling in deep layers of the dmPFC (Figures 1E,F). Importantly, this observation is consistent with the existence of abundant corticothalamic projections targeting the MD from cortical layers 5/6 (Gabbott et al., 2005).

### Connections Between the Insular and the Basolateral Complex of the Amygdala

To target IC-projecting neurons in BLA, we injected the AAV carrying a floxed hM4Di receptor expression cassette in BLA and the retrograde CAV2-Cre in IC (Figure 2A). Therefore, only amygdala cells projecting to the IC should be infected by both vectors and, thus, express mCherry and hM4Di. As shown in Figures 2B,C, we observed mCherry expression in the BLA and this expression was detected throughout the anteroposterior axis of the amygdala (between Bregma −2.04 and −3.48). Consistent with previous retrograde tracing studies (Sripanidkulchai et al., 1984), mCherry expression appeared to be greater in the basolateral (BL) and basomedial (BM) regions with less staining observed in the lateral amygdala however this was not formally quantified.

In a distinct group of rats, we reversed the location of the virus injections to study BLA-projecting neurons in the IC (Figure 2D). As shown in Figures 2E,F, we observed mCherry expression in most areas of IC, including agranular (dorsal and ventral) and dysgranular but little labeling in granular IC. We also observed some expression in the adjacent primary somatosensory cortex. mCherry expression was detected throughout the “gustatory IC” from Bregma +2.28 mm to 0.00 mm. This result is largely consistent with previous neuroanatomical tracing studies describing the connections from gustatory IC to the amygdala (see McDonald, 1998; Shi and Cassell, 1998).

### Cre-Independent Expression Using AAV8-hSyn-DIO-hM4Di-mCherry

**Figure 3:**
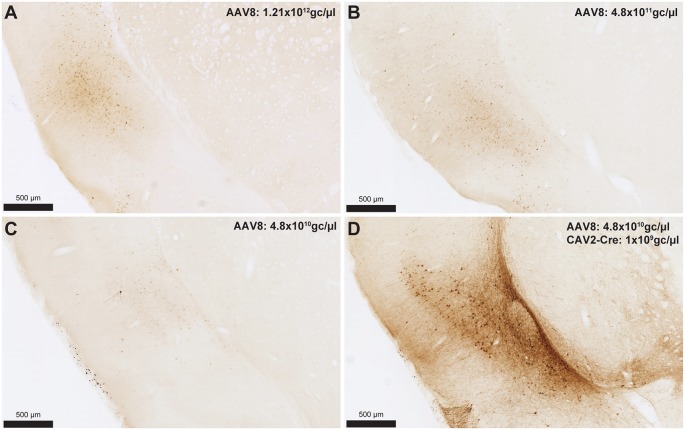
Cre-independent expression across decreasing titers **(A–C)**. AAV8-hSyn-DIO-hM4Di-mCherry was injected at three different titers: 1.21 × 10^12^ gc/μl (**A**; same titer as used in Figure 2), 4.8 × 10^11^ gc/μl **(B)** or 4.8 × 10^10^ gc/μl **(C)**. The expression of mCherry in the presence of Cre is shown in **(D)** using the weakest adeno-associated virus (AAV) titer (i.e., titer used in **C**). Images were captured using a Nanozoomer slide scanner (Hamamatsu Photonics) and the NDP.view 2.0^®^ freeware (Hamamatsu Photonics).

**Figure 4:**
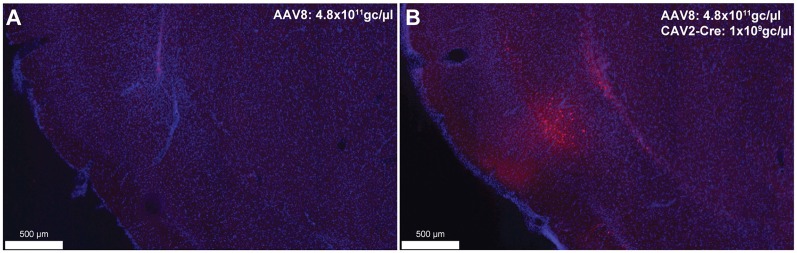
Cre-independent **(A)** and Cre-dependent **(B)** expression using the same titer as Figure 3B (AAV8-hSyn-DIO-hM4Di-mCherry at 4.8 × 10^11^ gc/μl). The inspection of these figures demonstrates that the extent of Cre-independent expression can be underestimated in fluorescence. Images were captured using a Nanozoomer slide scanner (Hamamatsu Photonics) and the NDP.view 2.0^®^ freeware (Hamamatsu Photonics).

To provide an estimation of the Cre-independent expression that can be observed using this AAV, three rats received an injection of AAV only in the IC at 1.21 × 10^12^ gc/μl (Figure 3A), 4.8 × 10^11^ gc/μl (Figure 3B) or 4.8 × 10^10^ gc/μl (Figure 3C). We also injected three rats with the weakest AAV dilution (4.8 × 10^10^ gc/μl) in the IC and the CAV2-Cre in the BLA (Figure 3D) to ensure that labeling of BLA-projecting IC neurons could still be obtained with a titer that minimized non-specific staining. As shown in Figures 3A–C, Cre-independent labeling was observed at all three titers. While this expression was not quantified, the amount of labeling seemed to decrease with the titer. It appears that at 4.8 × 10^10^ gc/μl minimal Cre-independent expression was observed (Figure 3C), while considerable Cre-dependent expression was preserved (Figure 3D). Finally, even at a titer where considerable leakage was observed in DAB amplification (4.8 × 10^11^ gc/μl; Figure 3B), immunofluorescence appeared insufficiently sensitive to reveal the Cre-independent expression (Figure 4), suggesting that this issue may be overlooked in similar studies that rely only on fluorescence.

## Discussion

Collectively, these data illustrate the use of the CAV2 vector to target neurons based on their anatomical connectivity in reciprocal thalamocortical and temporocortical pathways. In both circuits, the labeling of projection-defined neurons was found to closely match the currently known architecture of these connections. For instance, in the MD, most labeled cells occupied the lateral segment of the structure, consistent with the fact that it is the most lateral portion of that structure that projects to the dmPFC (Groenewegen, 1988; Alcaraz et al., 2016). In the dmPFC, all labeled cells appeared to be in deep layers, again in agreement that deep but not superficial cortical layers project back to the MD (Gabbott et al., 2005). Similarly, in the gustatory IC, the majority of staining was found in dysgranular and agranular areas with little staining in the granular region, as previously described (Shi and Cassell, 1998). It should be noted that we also observed extensive labeling of fibers in the central nucleus of the amygdala, which again aligns with previous studies (Shi and Cassell, 1998). As expected, in the BLA-to-IC pathway, neuronal staining was detected throughout the anteroposterior axis and there appeared to be more labeled neurons in the basolateral nucleus than in the lateral nucleus (Sripanidkulchai et al., 1984), although this observation was not quantified.

Off-target transgene expression or “leakage” has been observed for a variety of genetically coded neuroscience tools (for a review see Sjulson et al., 2016). Here, we illustrated this limitation in AAVs carrying DREADDs and the fluorescent protein, mCherry. As expected, this Cre-independent expression appeared to reduce with weaker virus titers (Figure 3). Such leakage may be an issue that is specific to AAVs as Cerpa et al report in this Research Topic that no such leakage is observed when using the novel CAV2-DIO-DREADD vector in wild-type rats at a similar titration and volume. It should also be noted that the CAV2-Cre vector can infect axons of passage, which may potentially lead to Cre expression in regions that do not project to the site of injection (Schwarz et al., 2015). If an AAV is injected in areas from which such axons arise, neurons that do not connect to the site of injections of the CAV2-Cre vector may express the gene carried by the AAV. Finally, as previously reported (Sjulson et al., 2016), we observed that relying on fluorescence may underestimate the extent of both specific and Cre-independent expression. Indeed, a more sensitive method (such as DAB amplification) may be required to fully appreciate the extent of the leakage. These observations highlight the need for careful selection of the optimal AAV dosage and the importance of explicitly acknowledging and illustrating transgene leakage, using sufficiently sensitive detection methods.

Finally, the interventions described here in the thalamocortical circuit have been previously found to produce a specific impairment in an adaptive decision-making task, showing that thalamocortical and corticothalamic pathways support complementary but dissociable aspects of decision processes (Alcaraz et al., 2018). Thus, using CAV2 to target projection-defined neurons enabled us to identify the directionality of the functional exchanges within neural circuits as an important feature. However, as the vector is known to rely on the presence of its CAR receptor for initial infection (Bru et al., 2010; Zussy et al., 2016), detailing the expression of this receptor at brain-wide levels and in various species would be useful to predict its efficacy in other brain regions and pathways.

## Data Availability Statement

The datasets generated for this study are available on request to the corresponding authors.

## Ethics Statement

Experiments were conducted in accordance with current laws and policies (French Council directive 2013-118, February 1, 2013 and European directive 2010-63, September 22, 2010). The experimental protocols received approval #5012053-A from the local Ethics Committee on December 7, 2012.

## Author Contributions

SM, RP, MW, and SP collected and analyzed the data. MW and SP wrote the manuscript. SM, RP, MW, and SP revised the manuscript and approved the final version.

## Conflict of Interest

The authors declare that the research was conducted in the absence of any commercial or financial relationships that could be construed as a potential conflict of interest.
